# The impact of iron supplementation on the preterm neonatal gut microbiome: A pilot study

**DOI:** 10.1371/journal.pone.0297558

**Published:** 2024-02-21

**Authors:** Matthew VanOrmer, Maranda Thompson, Melissa Thoene, Jean-Jack Riethoven, Sathish Kumar Natarajan, Corrine Hanson, Ann Anderson-Berry

**Affiliations:** 1 Department of Pediatrics, University of Nebraska Medical Center, Omaha, NE, United States of America; 2 Nebraska Center for Biotechnology, University of Nebraska-Lincoln, Lincoln, NE, United States of America; 3 Department of Nutrition and Health Sciences, University of Nebraska-Lincoln, Lincoln, NE, United States of America; 4 College of Allied Health Professions, University of Nebraska Medical Center, Omaha, NE, United States of America; University of Minnesota Twin Cities, UNITED STATES

## Abstract

**Objective:**

The gastrointestinal microbiome in preterm infants exhibits significant influence on optimal outcomes–with dysbiosis shown to substantially increase the risk of the life-threatening necrotizing enterocolitis. Iron is a vital nutrient especially during the perinatal window of rapid hemoglobin production, tissue growth, and foundational neurodevelopment. However, excess colonic iron exhibits potent oxidation capacity and alters the gut microbiome–potentially facilitating the proliferation of pathological bacterial strains. Breastfed preterm infants routinely receive iron supplementation starting 14 days after delivery and are highly vulnerable to morbidities associated with gastrointestinal dysbiosis. Therefore, we set out to determine if routine iron supplementation alters the preterm gut microbiome.

**Methods:**

After IRB approval, we collected stool specimens from 14 infants born <34 weeks gestation in the first, second, and fourth week of life to assess gut microbiome composition via 16S rRNA sequencing.

**Results:**

We observed no significant differences in either phyla or key genera relative abundance between pre- and post-iron timepoints. We observed notable shifts in infant microbiome composition based on season of delivery.

**Conclusion:**

Though no obvious indication of iron-induced dysbiosis was observed in this unique study in the setting of prematurity, further investigation in a larger sample is warranted to fully understand iron’s impact on the gastrointestinal milieu.

## Introduction

### An overview of the gut microbiome

The gastrointestinal microbiome bears considerable research interest due to its potential role in colorectal cancer [1–3], cardiovascular disease [4–6], inflammatory bowel conditions including *C*. *difficile* infection [7–9], and devastating diseases in preterm neonates such as necrotizing enterocolitis (NEC) [10–12]. Colonic bacteria are known to ferment carbohydrates to produce their cellular energy resulting in the production of short-chain fatty acids (acetate, butyrate, propionate) that are readily taken up by the epithelium to regulate human cellular activity [13,14] and gut inflammation [15,16]. The initial colonization of the neonatal gut by commensal microbes occurs during parturition at the latest via the transmission of maternal bacterial strains through exposure to the vaginal canal, skin, breastfeeding, or during the process of cesarean delivery [17,18]. Vaginal deliveries result in a higher load of beneficial strains such as *Lactobacilli sp*., while cesarean deliveries result in depleted microbial counts and increased colonization of pathogenic Proteobacterial strains (i.e. *Enterobacteriaceae)*, in addition to the delayed appearance and proliferation of commensal strains [19].

### The consequences of gut dysbiosis in preterm infants

Gastrointestinal dysbiosis in the preterm neonate during this period of extreme fragility can be devastating to the fragile tissue in these vulnerable patients’ gut. Perhaps chief among concerns regarding neonatal gut dysbiosis is NEC–a disease that comprises nearly 10% of all NICU deaths and bears a 30% mortality rate among infants born under 1500 grams [20]. Numerous studies implicate dysbiosis of the preterm gut microbiome–and the corresponding proliferation of pathologic, inflammation-inducing strains–in the etiology of NEC. *Enterobacteriaceae* in particular has been consistently shown to increase prior to NEC [21]. Further, the proliferation of Proteobacterial strains in the preterm neonate necessarily delays the typical dominance of beneficial strains (*Bifidobacteria* and *Lactobacilli*) with gut-protective outputs and anti-inflammatory action–a double-edged sword which exacerbates the risk of developing both NEC and subsequent morbidities.

### Iron’s clinical importance and impact on the gut microbiome

Iron is a vital nutrient in all living organisms and bears particular importance in humans for its role in hemoglobin synthesis, oxygen transport and storage, nucleic acid production, the electron transport chain, cell division, and gene expression [22,23]. Neonates rely heavily on adequate iron stores for appropriate development. Preterm neonates often miss out on crucial third-trimester iron loading [24] due to their early delivery and loss of direct connection to maternal iron stores–exacerbating their risk for poor neurodevelopmental outcomes. As such, dietary iron supplementation is recommended for preterm infants to avoid both the immediate and lifelong consequences of iron deficiency during this period–with the American Academy of Pediatrics encouraging a dose of 2mg/kg per day beginning at one month of age [25].

Iron’s specific impact on the gut microbiome in humans has been posited in the literature, but definitive human studies remain sparse. The majority of iron-oxidizing bacterial strains reside in the phylum Proteobacteria–which harbors many pathological and invasive bacterial strains. Iron-rich environments facilitate the proliferation of bacteria throughout the body, such that host immune mechanisms restrict iron access during periods of infection [26]. Iron supplementation trials in both African children [27] and infants/toddlers [28] each indicated dysbiosis stemming from dietary iron supplementation, with a proliferation of Protebacterial strains (*Enterobacteria* etc) and a reduction in *Lactobacilli* and *Bifidobacteria*. Of note, there is a paucity of research on the potential impact of iron on the gut microbiome in preterm infants. Given the evidence implicating dysbiosis in neonatal NEC risk, there is concern that routine iron supplementation may provide competitive advantage to the invasive pathogens–which our study sets out to clarify.

## Materials and methods

### Research setting and participant recruitment

An IRB-approved study (IRB #112-15-EP) obtained written parental consent from expectant and post-partum women presenting at Nebraska Medicine’s hospital Labor and Delivery Unit (Omaha, NE) to enroll their newborn infants in an analytic observational cohort study. Eligibility criteria included mothers ≥19 years of age with infants born ≤34 weeks corrected gestational age (CGA) and admitted to Nebraska Medicine’s Neonatal Intensive Care Unit (NICU) who intended to exclusively provide breast milk their infants (either *per os* or via a nasogastric tube)–the use of pasteurized donor human milk and parenteral nutrition in infant participants was acceptable for enrollment, with maternal intent to exclusively breastfeed. There were no additional exclusion criteria. Patients in this cohort were recruited between October 1^st^, 2018 and June 30^st^, 2019. Due to the nature of the patient population in this study, critically-ill infants were eligible for enrollment; as such, study procedures were terminated immediately in the event of neonatal death. Enrollment must have occurred no later than the infant’s fifth day of life, to ensure timely collection of initial biospecimens.

### NICU nutrition practices

Enteral nutrition management among enrolled preterm infants were initiated via nasogastric tube as soon as medically appropriate, preferably within the first day of life targeting 30-35mL/kg/day. Trophic feeds at this volume continued for 48 hours in infants born <28 weeks gestational age; those born >28 weeks gestational age did not receive trophic feeds but rather enteral nutrition volume was increased daily by 30-35mL/kg as tolerated toward a goal of 150mL/kg/day. Upon reaching 50-60mL/kg/day, human milk was fortified to 24kcal/oz using non-acidified liquid human milk fortifier, followed by liquid protein fortification subsequent to 24 hours of tolerated caloric fortification. When mothers own milk was unavailable, pasteurized donor milk purchased from a donor human milk bank was used as a supplement with parental consent, with similar fortification strategies to mother’s own milk. Probiotic supplements (0.5 grams/day) containing five bacterial strains (Bifidobacterium breve, Bifidobacterium longum, Bifidobacterium infantis, Bifidobacterium bifidum, and Lactobacillus rhamnosus) were initiated once enteral feeding volumes reached 48mL/day. Daily enteral iron fortification beginning at 14 days of life occurred in any infant receiving at least half of their enteral feeding volume as mother’s own milk or pasteurized donor human milk, via ferrous sulfate supplementation at 3mg/kg/day (range 2-4mg/kg/day).

### Specimen collection and analysis

Infant stool specimens were collected three times during NICU admission–once each during the first, second, and fourth weeks of life (WOL). This schedule of stool specimen collection was designated to capture specimens both before and after the initiation of standard-of-care iron supplementation in our NICU practice beginning at the 14^th^ day of life, while also providing duplicate measurements during the first two weeks of life to correct for dramatic gastrointestinal microbiome changes immediately after birth. Stool specimens were captured during typical infant cares by clinical staff directly from the infant’s diaper using study team-provided PSP Spin Stool DNA Plus collection tube containing DNA stabilizing solution, which renders DNA specimens stable at room temperature for up to 90 days. Specimens were stored at -20°C within 48 hours of bedside collection to maximize the longevity of DNA stability, and specimens were subsequently processed in bulk adhering to the manufacturer’s protocol. Sequencing the resulting DNA took place using an Illumina MiSeq platform targeting the V4-V1 primers of the 16S rRNA gene, including PCR amplification, two rounds of PCR clean-up passes, and library quantification and normalization. Sequence generation was conducted in collaboration with the University of Nebraska Medical Center’s Genomics Core Facility.

### Bioinformatics and statistical considerations

[29–32] Generated sequences were assessed for quality and demultiplexed using Illumina software (MiSeq Control Software version 2.6) according to the manufacturer’s guidelines. After the demultiplexing step, bioinformatics analyses were performed following the Bioconductor workflow for microbiome data analysis by Callahan et al. [33] using R software [34]. In brief, for the initial steps, the R package DADA2 [29] (version 1.18.0) was used. These steps include: denoising, chimera removal, clustering of high-quality sequencing reads to infer amplicon sequence variants (ASV), and calculation of ASV counts per sample. A naïve Bayes taxonomy classifier [30] classified each ASV against the SILVA 138.1 reference database [35] to construct a taxonomy table, while MAFFT [31] (version 7.407) and FASTTREE [32] (version 2.1.11) programs constructed a phylogenetic tree. Taxa abundances at the phyla and genera level were normalized with the total sum scaling normalization method dividing each ASV count by the total library size to yield their relative proportion of counts for each sample. Shannon Alpha diversity was calculated with the R packages phyloseq [36] (version 1.34.0) and vegan [37] (version 2.6.2). Bioinformatics analysis of sequences specimens was conducted by the University of Nebraska Lincoln’s Bioinformatics Core. Sequence data for this cohort is accessible via BioProject (PRJNA1019326).

Descriptive statistics were generated for both study exposure and outcome measures as well as demographic characteristics such as infant sex and gestational age at birth; medians, interquartile ranges, minimums, and maximums were generated for continuous variables, and frequencies and percents were utilized for categorical variables. Ratios were utilized to quantify relative abundances of various bacterial strains as a proportion of total gastrointestinal bacteria colonization. The average of pre-iron collection timepoints (i.e. WOL 1 and WOL 2) was generated for continuous variables in order to mitigate dramatic early life differences in gastrointestinal composition and was used in direct comparison to post-iron values in certain statistical analyses (i.e. WOL 4 values)–average was utilized in this fashion as opposed to median due to the small number of values being aggregated; in some cases, only two. Exposure and outcome measures were assessed for normality, and the Wilcoxon Signed Rank tests were used as appropriate to compare averaged pre-iron initiation values to post-iron values. As Shannon Diversity is normally distributed, paired sample T-tests were used to compare pre- and post-iron diversity levels. Repeated measures ANOVA (Friedman’s test for non-normally distributed variables) were also used to assess differences in continuous variables across each of the three collection timepoints. Independent sample T-tests or the Mann-Whitney U test were utilized to assess differences in aggregate participant measures based on categorical delineators (i.e. sex, season of specimen collection, etc). Fisher’s exact test associated dichotomous categorical variables, and Spearman correlations to relate continuous measures. A p-value was considered statistically significant in all analyses. All statistical analyses were conducted in IBM SPSS Statistics version 28 [38].

## Results

### Participant demographics and baseline characteristics

A total of 13 mothers provided informed consent for their newborns to participate in this study between November of 2018 and June of 2019, resulting in 14 preterm neonates enrolled for stool sample collection (12 singleton deliveries and one set of twins). One neonatal participant passed away resulting from their critical condition during the study period. A total of 39 stool specimens were collected from enrolled infants based on our repeated measures schedule, with 34 specimens suitable for quantification of microbiome composition. The median gestational age at birth of the infants was 29.43 weeks (IQR 27.00–30.86), with the most-preterm infant in this cohort born at 23.57 weeks gestation. Our cohort had an even split of male and female infants, with seven in each group. Vaginal deliveries accounted for five of the 14 infants (37.7%), with cesarean deliveries accounting for the remaining 9 infants (64.3%). The racial and ethnic makeup of infant participants included 6 (42.9%) White infants, 3 (21.4%) Black infants, 4 (28.6%) Hispanic/Latino infants, and one (7.1%) infant of Other/Unknown race.

### Dynamics of the gut microbiome across NICU admission

Predominant phyla quantified in this cohort included Firmicutes, Bacteroidetes, Proteobacteria, and Actinobacteria–trace amounts (<0.001%) of Verrucomicrobia, Cyanobacteria, and Tenericutes were observed in select samples but were excluded from analyses due to their extremely small absolute and relative abundances. ASVs belonging to the genera *Lactobacilli* and *Bifidobacteria* were appropriately categorized as such and quantified in both absolute abundance and relative abundance of total microbiome makeup. Median phyla relative abundance was 31.23% *Firmicutes*, 0.05% *Bacteroidetes*, 20.72% *Proteobacteria*, and 29.81% *Actinobacteria*. At the genus level, 13.31% of strains belonged to genus *Lactobacilli*, and 29.77% of strains belonged to genus *Bifidobacteria*.

Comparisons of each phylum and genus’s mean relative abundance based on dichotomous participant characteristics are presented in **Table 1**. In short, no significant differences in any phyla or genera’s mean relative abundance were observed between participant sex (all *p*-values >0.40) or delivery mode (Firmicutes, Bacteroidetes, and Proteobacteria *p*-values >0.30)–though Actinobacteria and its constituent *Bifidobacteria* mean relative abundance approached a significant difference between delivery modes, with a trend toward higher abundance among vaginally-delivered infants versus cesarean sections (*p* = 0.07).

**Table 1:** Mann-Whitney U test of mean relative abundance across all weeks of life between dichotomous participant characteristics.

	Sex			Delivery Mode		
	*Female*	*Male*	*p-value*	*Vaginal*	*Cesarean*	*p-value*
**Firmicutes**	30.51%	35.16%	0.95	31.94%	30.51%	0.74
**Bacteroidetes**	0.05%	0.04%	0.41	0.05%	0.04%	0.46
**Proteobacteria**	30.04%	11.41%	0.85	5.59%	46.89%	0.32
**Actinobacteria**	30.24%	29.38%	0.75	51.25%	28.29%	0.07
***Lactobacilli***	15.63%	11.68%	0.48	15.94%	7.97%	0.21
***Bifidobacteria***	30.17%	29.37%	0.75	51.18%	28.29	0.07

Mean phyla relative abundances were also related to infant CGA (Corrected gestational age) using Spearman correlations. No significant relationships were observed between mean phyla relative abundances and CGA, though similarly to differences by delivery mode Actinobacteria and *Bifidobacteria* relative abundance approached significant positive correlations with CGA (R = 0.52, *p* = 0.06). All Spearman correlations between phyla and genera relative abundance and CGA are presented in **Table 2.**

**Table 2:** Spearman correlations between mean relative abundance across all weeks of life and infant birth CGA.

	Spearman’s R	*p*-value
**Firmicutes**	0.16	0.59
**Bacteroidetes**	-0.08	0.78
**Proteobacteria**	-0.24	0.40
**Actinobacteria**	0.52	0.06
***Lactobacilli***	-0.02	0.95
***Bifidobacteria***	0.52	0.06

**Fig 1:**
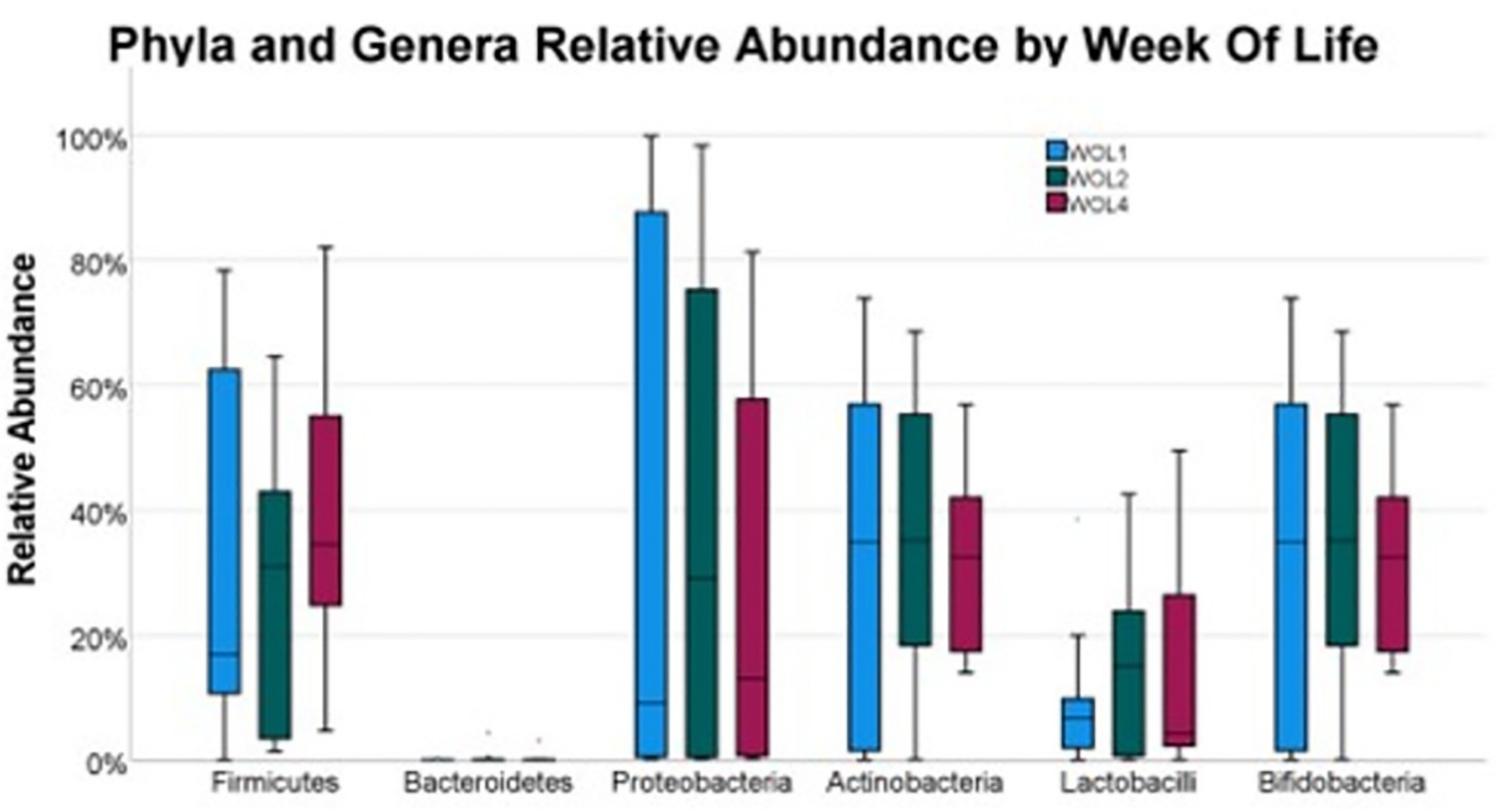
Phyla and genera relative abundance by week of life.

Friedman’s test was utilized to assess any differences in the distribution of each individual phylum and genus’s relative abundance across the three-sample collection timepoints–no significant differences in distribution between WOL1, WOL 2, and WOL 4 were observed for any of the four major phyla (Firmicutes *p*-value = 0.42, Bacteroidetes *p*-value = 0.51, Proteobacteria *p*-value = 0.97, Actinobacteria *p*-value = 0.88) nor either genus of interest (*Lactobacilli* and *Bifidobacteria p* = 0.88). Pre-iron initiation relative abundance measurements were averaged together and compared with the WOL 4 relative abundance using Wilcoxon Signed Rank tests to further investigate what effect iron initiation has on the gut microbiome. No significant differences in relative abundance distributions were observed between pre- and post-iron measurements (All p-values >0.05). The relative abundance distributions by week are represented in **Fig 1**.

#### Microbial diversity and seasonal shifts in the neonatal gut

The median Shannon diversity over the study period for all subjects was 1.90 (IQR: 1.82–2.13). Shannon diversity did not differ based on infant sex (*p* = 0.99) or infant delivery mode (*p* = 0.88), nor did it significantly correlate with infant CGA (Spearman’s R = 0.47, *p* = 0.09). One-way ANOVA of Shannon diversity measurements at our three time points revealed statistically significant differences in distribution over time (F = 8.84, *p* = 0.003), with pairwise comparisons revealing significantly lower Shannon diversity in WOL 1 versus WOL 2 (1.36 vs 1.78, *p* = 0.02), and WOL 1 versus WOL 4 (1.36 vs 2.02, *p* = 0.005). Shannon diversity prior to iron initiation was subsequently averaged and compared to WOL 4 Shannon diversity using a paired sample T-test, which indicated a statistically significant difference in pre- and post-iron Shannon diversity (1.71 vs 2.02, *p* = 0.012).

An unintended result of our recruitment schedule was a cohort of patients divided into distinct seasons of delivery–eight infants (57.1%) were born between November-December (i.e Winter), and six (42.9%) infants were born between March-May (i.e. Spring), allowing us to conduct a preliminary evaluation of seasonal differences in gastrointestinal colonization. Of note, no difference in infant CGA was observed between Winter and Spring deliveries (30.2 vs 28.6 weeks, *p* = 0.48) and no association existed between delivery mode and season of birth (Fisher’s exact *p* = 0.58). Mean phyla-level relative abundance, mean genera-level relative abundance, and mean Shannon diversity across each collection timepoint were compared between Winter and Spring delivery groups using the Mann-Whitney U test, with numerous significant differences observed and a number approaching significance–these findings are displayed in **Table 3.**

**Table 3:** Mann-Whitney U comparisons of gastrointestinal characteristics between winter and spring deliveries.

	Winter	Spring	*p*-value
**Firmicutes**	38.21%	18.67%	0.04*
**Bacteroidetes**	0.05%	0.04%	0.52
**Proteobacteria**	5.26%	59.52%	0.07
**Actinobacteria**	41.68%	16.94%	0.05*
***Lactobacilli***	15.29%	2.68%	0.09
***Bifidobacteria***	41.54%	16.94%	0.05*
**Shannon Diversity**	1.90	1.93	0.19

* Indicates p-value <0.05.

## Discussion

The overall phylogenetic breakdown of our cohort’s gut microbiome largely resembles previous characterizations of the preterm neonatal gut microbiome [39–41], though with lower abundance of Bacteroidetes. This is likely due to the exclusively human-milk fed recruitment in our cohort versus previous studies characterizing both human-milk fed and formula fed infants–the latter of which exhibit lower abundance of Actinobacteria. Further, supplementation with numerous *Bifidobacteria* strains (of the phylum Actinobacteria) in the form of probiotics likely accentuated the abundance of Actinobacteria in our cohort. We also observed trends toward significance that align with characteristic influences on infant gut microbiome composition–namely, the role of both gestational age and delivery mode. In our study, Actinobacteria had lower average relative abundance among infants delivered via cesarean section than their vaginally delivered counterparts (51.25% vs 28.29%, *p* = 0.07), and Actinobacteria relative abundance also exhibited a moderated positive correlation with gestational age, though both only approached statistical significance (R = 0.518, *p* = 0.06). Korpela et al. similarly demonstrated the effect of gestational age on Actinobacteria abundance, highlighting that one of the final stages of neonatal gut microbiome development is the rapid proliferation of *Bifidobacteria* at 30 weeks post-menstrual age [42]. Further, many previous studies have demonstrated differences in *Bifidobacteria* relative abundance between vaginal and cesarean deliveries [43–47], with consistently lower abundance in the latter.

Our study observed notable significant differences in bacterial colonization based on infant season of delivery. Infants born in the Spring had precipitous drops in relative abundance for Firmicutes and Actinobacteria (corresponding to drops in *Lactobacilli* and *Bifidobacteria* relative abundance), with concomitant and sizable increase in Proteobacteria relative abundance–up from 5% in Winter deliveries to 59% in Spring deliveries (though only approaching significance). It is challenging to precisely identify the cause of these seasonal differences in gut microbiome composition, but one explanation is variation in maternal diet patterns resulting in subtle changes in vertical transfer of bacterial strains from mother to infant. Davenport et al. have demonstrated significant differences in both Firmicutes and Actinobacteria across seasons in adults, with higher abundance in summer versus winter timepoints–mirroring the results observed in our cohort [48]. Another intriguing possibility is variation in the environmental microbiome of our NICU setting–work by Brooks et al. have indicated notable overlap between admitted neonate’s gut microbiome and the surrounding room’s bacterial colonization across admission, with some instances of room colonization shifts appearing prior to patient microbiome changes [49,50]. Further, Rozé et al. have demonstrated the impact of NICU care practices on gut microbiome composition [51], reinforcing the possibility of environmental influences that change across seasons carrying over into neonatal gut microbiome changes. Although we did not observe any difference in CGA between seasonal groups, nor was season associated with delivery mode or infant sex, we cannot definitively rule out the possibility of clinical differences between Winter and Spring infants in our relatively small sample.

This study was conceptualized in part due to findings from two interventional trials of iron supplementation in sub-Saharan African infants and children from Tang et al. [52] and Zimmerman et al. [27]–each of which demonstrated gastrointestinal dysbiosis in those supplemented with enteral iron. Importantly, these studies evaluated the effect of iron supplementation on infants at least six months of age (i.e. well past the initial post-birth stage of gastrointestinal microbiome development) and children between 6- and 12-years old, and each in a resource-poor rural African population. A handful of other studies–primarily in anemic African infants and toddlers–exist evaluating iron’s role in gut dysbiosis [28,53]; however, to our knowledge only one other study has evaluated iron’s role on the preterm infant gut microbiome, which was published after the completion of our cohort’s recruitment. In their study, Ho et al. recruited 80 very low birth weight infants and collected stool samples over the first two months of life, subsequently assessing the makeup of their gut microbiome based on iron dosage–which ranged from 3mg/kg/day-6mg/kg/day [54]. They observed significantly higher abundance of Proteobacteria only in the highest iron dosage group (i.e. ≥6mg/kg/day) with a corresponding drop in Shannon diversity. Ho et al. were able to recruit to sufficiently power their study and generate findings that potentially indicate a role for iron in the neonatal gut microbiome–namely, increased Proteobacteria abundance and reduced diversity. However, the differences they observed were almost exclusive to the ≥6mg/kg/day group of infants, which also skewed younger (average of 26.2 CGA), had a high prevalence of mixed feeding participants (46%), and were almost exclusively cesarean deliveries (85%). Further, ≥6mg/kg/day iron dosing is roughly twice that of our NICU clinical practice, making direct comparison to our cohort challenging. It is possible that at this high per-weight dosage excess iron is sufficiently disruptive to the gut microbiome, but at lower doses no issues arise. With that said, Ho et al.’s study is an important work further clarifying the impact of iron supplementation on the preterm infant gut microbiome, perhaps identifying a dosage level that elicits dysbiosis which we were not able to identify in our patient population due to more restrained dosing practices.

## Conclusions

The primary limitation of this study is low sample size. Despite our repeated measures sampling strategy, only 14 preterm infants were enrolled in this study with a total of 39 stool specimens available for analysis. Low sample size limited our ability to account for numerous confounding variables in multivariate analyses–including the considerable confounding effect of antibiotic use in these patients and the evaluation of proportion of mother’s own milk vs alternative substrates (i.e. donor milk). Our findings must be considered in the context of our low N and lack of adjustment for relevant confounders, but present additional data regarding the state of preterm infant gut microbiome constituents over the course of NICU admission. Further, use of the Mann-Whitney U test in low-N relative abundance assessment bears the risk of poor power in small N studies [55]–which may have resulted in unobserved differences. That said, the dearth of evidence regarding iron’s effect on the preterm infant gut microbiome–particularly among very preterm infants–results in this small study still contributing valuable information to the wider body of data available on this potential effector of gastrointestinal risk. Additional limitations include the lack of fecal iron concentration measurement as a means of assessing direct correlations between microbial abundance and excess iron available in the colon. Finally, our sampling schedule was one of convenience to ensure equal collection among participants with highly varied admission durations.

Our study evaluated what impact initiation of routine enteral iron supplementation in preterm infants had on gastrointestinal dysbiosis and observed no significant shift in gut microbiome composition in pre- and post-iron specimens. We observed changes in gastrointestinal colonization based upon infant season of delivery, with a shift toward pathogenic strains in infants born in Spring vs Winter. Further study is warranted to fully understand the role iron may play in the competitive balance of the gastrointestinal microbiome, especially in the highly vulnerable population of preterm infants.
